# Effects of sub-lethal concentrations of lindane on histo-morphometric and physio-biochemical parameters of *Labeo rohita*

**DOI:** 10.1371/journal.pone.0304387

**Published:** 2024-07-05

**Authors:** Gulnaz Afzal, Hafiz Muhammad Ali, Tariq Hussain, Shujaat Hussain, Muhammad Zishan Ahmad, Adeeba Naseer, Rehana Iqbal, Jawaria Aslam, Ahrar Khan, Mohamed Farouk Elsadek, Bandar M. Al-Munqedhi, Riaz Hussain

**Affiliations:** 1 Department of Zoology, The Islamia University of Bahawalpur, Bahawalpur, Pakistan; 2 Faculty of Veterinary and Animals Sciences, The Islamia University of Bahawalpur, Bahawalpur, Pakistan; 3 Department of Basic Sciences, College of Veterinary and Animal Science, Jhang, Pakistan; 4 Faculty of Veterinary and Animals Sciences, PMAS Arid Agriculture University, Rawalpindi, Pakistan; 5 Zoology Division, Institute of Pure and Applied Biology, Bahauddin Zakariya University, Multan, Pakistan; 6 Bahawalpur Medical and Dental College, Bahawalpur, Pakistan; 7 Shandong Vocational Animal and Veterinary Science College, Weifang, China; 8 Department of Biochemistry, College of Science, King Saud University, Riyadh, Saudi Arabia; 9 Department of Botany and Microbiology, College of Science, King Saud University, Riyadh, Saudi Arabia; University of Ibadan Faculty of Science, NIGERIA

## Abstract

Lindane is a broad-spectrum insecticide widely used on fruits, vegetables, crops, livestock and on animal premises to control the insects and pests. The extensive use of pesticides and their residues in the soil and water typically join the food chain and thus accumulate in the body tissues of human and animals causing severe health effects. The study was designed to determine the toxicity effects of sub-lethal concentrations of lindane on hemato-biochemical profile and histo-pathological changes in Rohu (*Labeo rohita*). A significant increase in the absolute (p<0.05) and relative (p<0.05) weights was observed along with severe histo-pathological alterations in liver, kidneys, gills, heart and brain at 30μg/L and 45μg/L concentration of lindane. A significant (p<0.05) decrease in RBCs count, PCV and Hb concentration while a significant (p<0.05) increased leukocytes were observed by 30μg/L and 45μg/L concentrations of lindane at 45 and 60 days of the experiment. Serum total protein and albumin were significantly (p<0.05) decreased while hepatic and renal enzymes were significantly (p<0.05) increased due to 30μg/L and 45μg/L concentrations of lindane at days-45 and 60 of experiment compared to control group. The observations of thin blood smear indicated significantly increased number of erythrocytes having nuclear abnormalities in the fish exposed at 30μg/L and 45μg/L concentrations of lindane. ROS and TBARS were found to be significantly increased while CAT, SOD, POD and GSH were significantly decreased with an increase in the concentration and exposure time of lindane. The results showed that lindane causes oxidative stress and severe hematological, serum biochemical and histo-pathological alterations in the fish even at sub-lethal concentrations.

## Introduction

Lindane is a broad-spectrum organo-chlorine insecticide that act as an endocrine disruptor and is used to control the insects and pests of rice, cotton and soyabean [1, 2]. Lindane is also widely sprayed on fruits, vegetables, crops, animals and the livestock farms at worldwide for the last seven decades [3, 4]. As a result of their extensive applications, through their easy entry into the aquatic ecosystem as “runoff”, these insecticides cause serious harm to the non-target organisms, particularly fish [5–7]. The exposure to these pollutants cause bio-diversity instabilities like food web damaging, habitat loss and multiple organ dysfunctions resulting in poor performance of various organisms [8–12]. Moreover, accidental exposure in marine, freshwater and terrestrial environments to adverse external toxins not only cause fatal outcomes but also reduce the life span of several non-target species [13–19]. Furthermore, the aquatic species are additionally at enormous risk relative to the terrestrial organisms since industries and agriculture wastes are directly and easily transferred to the water bodies.

Insecticides are usually not biodegradable and tend to reside in the soil and water for years. The reported half-life of lindane in soil and water is 708 and 2292 days, respectively [20, 21]. The soil residues of lindane typically join the food chain and accumulate in the fat tissues of human and animals causing significant health hazards like mutagenic, genotoxic and teratogenic effects [22]. Epidemiological and agricultural health studies have confirmed that the exposure to lindane may be associated with cancers of breast, prostate, lung, stomach, colon, rectum and bladder in humans [23–25]. Due to environmental tenacity and bio-accumulation potential of lindane along with its both α and β isomeric chains, it was encompassed in the Stockholm Convention list of persistent organic pollutants (POPs) and thus, was banned or strictly circumscribed [26–28]. Since 26 March 2019, the Chinese Ministry of Ecology and Environment has prohibited the production, circulation, application, import and export of lindane in order to enforce the Stockholm Convention on POPs [29].

Due to its low cost and effectiveness in pest control, lindane is still used for agricultural and public health purposes in some developing nations including Pakistan [30, 31]. India is currently the largest lindane producer and consumer in the world [32, 33]. Aquatic organisms are under the influence of different environmental stress factors including pesticides [34, 35] and fish are considered the most vulnerable species among the aquatic organisms to various endocrine disrupting chemicals and hence, are valuable biomarkers for monitoring the quality of the aquatic world. The excessive use of pesticides not only cause atmospheric and soil pollution but also access the water bodies via surface water draining, spray drift and seepage through ground water resources and ultimately cause negative effects to the aquatic environments [36]. The absorption of lindane into aquatic animals and dermal interaction with infected water causes alterations in the behavior and physical parameters of fish like loss of locomotion and habits of swimming. Since, it bio-accumulates in the microorganisms, invertebrates, fish, birds and mammals, hence, is a threat to a wide range of ecosystems [37] and also equally a threat to the human health through the food chain.

Hematological and biochemical parameters could be used as useful bio-indicators in the fish as are well-known target tissues of pesticide toxicity [38–41]. Moreover, the alterations in the biochemical parameters like proteins, enzymes and glucose are used to assess the physiological changes in the aquatic environments due to stress conditions [42]. In has been reported that lindane is very harmful to the climate, humans and all types of wildlife including terrestrial and aquatic animals. The toxicity effects of lindane on hematological and biochemical parameters in *Cyprinus carpio L* [43], reproduction [44] and gene expression [21] in *Danio rerio* as well as behavior [27] and oxidative stress [45] in *Cyprinus carpio* has been documented. However, the toxicity of lindane in freshwater fish has very scarcely documented and in the few studies, the authors studied the hiso-pathological effects of lindane only on liver, gills and kidney of *Labeo rohita* [46], *Etroplus maculates* [47], *Aspidoparia morar* [48], *Mugil cephalus* and *Cyprinus carpio* [49], *Solea senegalensis* [50], *Sparus aurata* [51], on intestine of *Ctenopharyngodon idella* [7] and on haemopoietic tissue of *Labeo boga* [52]. Similarly, few hematological alterations have been found by lindane in *Aspidoparia morar* [53], *Cyprinus carpio* [43] and *Etroplus maculates* [47]. Therefore, the current study was planned to comprehensively determine the toxicity of lindane at sub-lethal concentrations on complete hemato-biochemical profile and histo-pathological parameters of *Labeo rohita* and hence, to urgently consider and manage the hazardous issues of this pesticide.

## Materials and methods

### Reagents and chemicals required

Lindane of analytically pure (>99.0%) grade was obtained from the commercial market, Lahore, Pakistan. All other chemicals were of analytical grades and were purchased from Sigma Aldrich (St. Louis Missouri, USA) and Merck (Germany). Serum biochemical kits were purchased from Randox Company (Pvt.) Pakistan.

### Fish management and experimentation

Rohu (*Labeo rohita*) of about the same weight (150–160g) were procured from the fish breeding center, Bahawalnagar, Pakistan. The experiments were conducted in the laboratory of Department of Life Sciences (Zoology), The Government Sadiq College Women University, Bahawalpur, Pakistan. All the animal experimentations were approved by the Institutional Review Board of The Government Sadiq College Women University, Pakistan (Protocol No. GSCWU-529).

The fish were placed in the standard housing conditions in tap water glass aquaria for 15 days for acclimatization. The metrics of water chemistry was determined before the start of the trial and maintained during the whole duration of the experiment, to keep a comfortable environment for the fish. The water of the aquarium was maintained at a temperature of 20.5°C, pH at 7.43 with a salinity level at 0.5ppt, total dissolved solids (0.43ppt), electrical conductivity (1.68mS/cm), ammonia (0.5mg/L) and dissolved oxygen at a level of 6.32ppm. Commercially available fish feed containing crude protein (22% protein) and groundnut cake was offered to the fish in the form of pellets (2–3% of body weight) at twice every day. To remove the residual feed and fecal materials from the aquaria, the water was replaced on regular basis to keep the environment clean and healthy, with a fresh addition of lindane to maintain a constant level of this pesticide during the exposure period.

At the start of experiment, the fish (n = 80) were randomly divided into four groups (20 / group). Group A was kept as control, while the fish of groups B, C and D were subjected to 15μg/L, 30μg/L and 45μg/L of lindane, respectively, for a duration of 60 days. The blood samples (2.5ml) were obtained from the caudal vein of each fish by using 26 gauge disinfected hypodermic needle on days-15, 30, 45 and 60 [16].

### Body mass, organ weight and histopathology

At days-15, 30, 45 and 60, five fish from each group were euthanized by using clove oil (4mg/L of clean water) to minimize the stress. The body mass (g) of the fish was determined and liver, kidneys, gills, heart and brain were collected and weighed by using an electric weighing balance to determine the absolute weight (g) of these organs. The relative organ weight (g) was determined as organ weight (g) / body weight of fish (g) × 100.

For histo-pathological examinations, 0.5cm tissue sample of each collected organ was preserved in 10% neutral buffered formalin solution and then processed by paraffin sectioning technique and 4–5μm thick histological sections were stained by Hematoxylin and Eosin staining technique [8, 54–56]. All the observations are an average of three independent slides for each tissue.

### Hematological studies

Approximately 2.5ml of blood was collected from the caudal vein of each fish by using sterile 26-gauge hypodermic needle. The collected blood (0.5ml) was immediately placed in anti-coagulants coated vacutainers. The hematological parameters like RBCs count, total (TLC) and differential (DLC) leukocytic count [57], as well as percent of hematocrit, hemoglobin (Hb) quantity and total protein [8, 58, 59] were measured at days-15, 30, 45 and 60 of the experiment, as previously described.

### Serum biochemical studies

Serum was separated from the remaining blood (2.0ml) of each fish, collected in anti-coagulant free containers and preserved at -20ºC until further serological analysis [60, 61]. The chemistry analyzer was used to test the serum biochemical parameters including total proteins, albumin, alanine aminotransferase (ALT), aspartate aminotransferase (AST), alkaline phosphatase (ALP), lactate dehydrogenase (LDH), urea and creatinine by using the commercially available kits of Randox (Pvt) Ltd.

### Morphological and nuclear changes in RBCs

A fine, thin blood smear was prepared from the fresh blood of each fish for observations of morphological and nuclear changes in the red blood cells (RBCs). All blood films were dried instantly, fixed with absolute alcohol, stained with Giemsa stain and studied at 1000X under light microscope [62]. Approximately 1500 RBCs from each fish were examined under light microscope at oil immersion lens [16].

### Determination of oxidative stress and anti-oxidant enzymes status

Different parameters of oxidative stress i.e. thiobarbituric acid reactive substances (TBARS) [63] and reactive oxygen species (ROS) [64, 65] and anti-oxidant enzymes including peroxidase (POD) [66], catalase (CAT), superoxide dismutase [67] and reduced glutathione (GSH) [67] were determined.

### Statistical analysis

The data were presented as mean ± S.E. (standard error) of three independent replicates. All the data of body weight, organ weight, nuclear abnormalities, hematological parameters and parameters of serum biochemistry in the treated groups compared to control were subjected to one-way analysis of variance (ANOVA) (version 20) followed by Tukey’s post-hoc test by using IBM SPSS^®^ statistics software. The level of significance was set at p < 0.05.

## Results

### Clinical signs of lindane exposure

Different clinical signs including loss of equilibrium, black spots on surface of the body, fin tremors, jerking movements, increased mucus secretions, surface breathing and swimming on one side were observed in the fish exposed to higher concentrations of pesticide at days-45 and 60 of the experiment (Table 1).

**Table 1:** The severity of various clinical signs in the fish exposed to different concentrations of lindane.

Days	Clinical signs	Concentrations of lindane (μg/L)			
		0μg/L	15μg/L	30μg/L	45μg/L
**45**	Loss of equilibrium	-	+	+	+++
	Black spots on surface of body	-	+	++	++
	Fin tremors	-	+	++	++
	Jerking movements	-	+	++	+++
	Increased mucus secretions	-	+	++	+++
	Increased surface breathing	-	+	++	++
**60**	Loss of equilibrium	-	+	++	+++
	Black spots on surface of body	-	+	+++	+++
	Fin tremors	-	+	++	+++
	Jerking movements	-	+	+++	+++
	Increased mucus secretions	-	++	+++	++++
	Increased surface breathing	-	+	+++	+++
	Swimming on one side	-	++	+++	+++

Absent = -, Mild = +, Moderate = ++, Severe = +++, Very severe = ++++

### Absolute organ weight and relative organ weight

**Fig 1:**
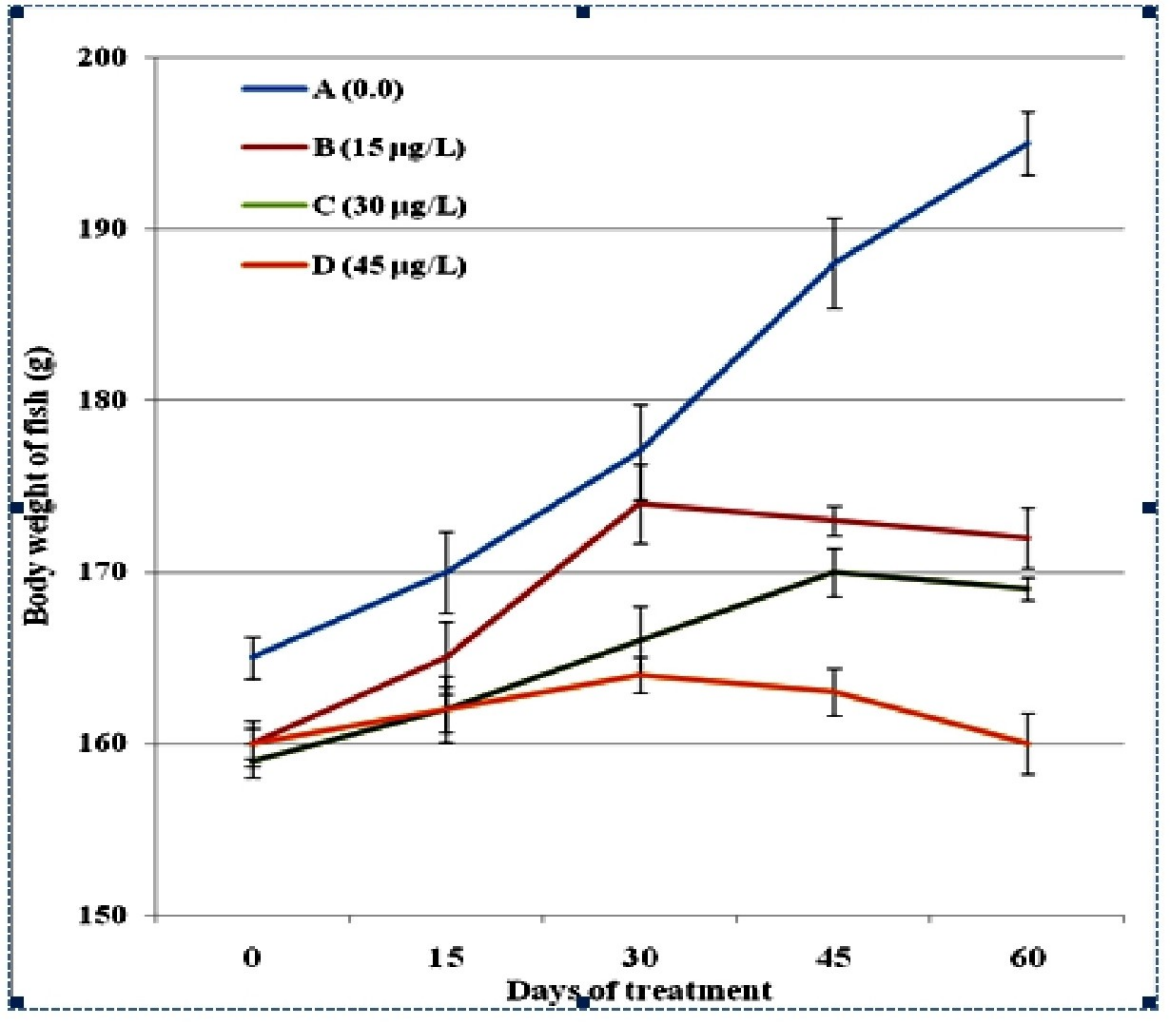
Absolute body weight (g) of fish at different time intervals (days) exposed to different concentrations of lindane. The body weight of fish exposed to higher concentration (45μg/L) of lindane was reduced significantly (p<0.05) in comparison to the control group at day-60 of the trial. Group A = control, B = 15μg/L, C = 30μg/L, D = 45μg/L of lindane.

**Fig 2:**
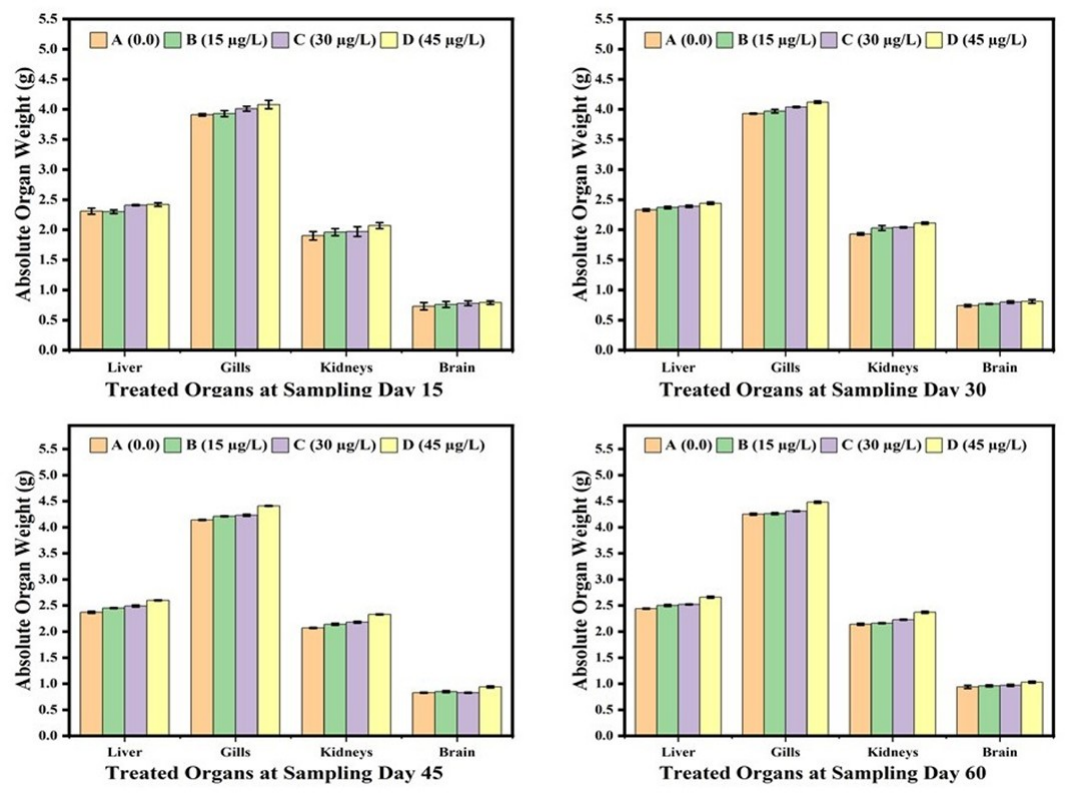
Absolute weights (g) of different organs of fish exposed to different concentrations of lindane at different time intervals / days. The group exposed with 45μg/L of lindane showed a significant (p<0.05) increase in the absolute weights of liver, kidneys and gills compared to control group at days-45 and 60 of the study while there observed no change in the weight of brain at different time intervals. Group A = control, B = 15μg/L, C = 30μg/L, D = 45μg/L of lindane.

**Fig 3:**
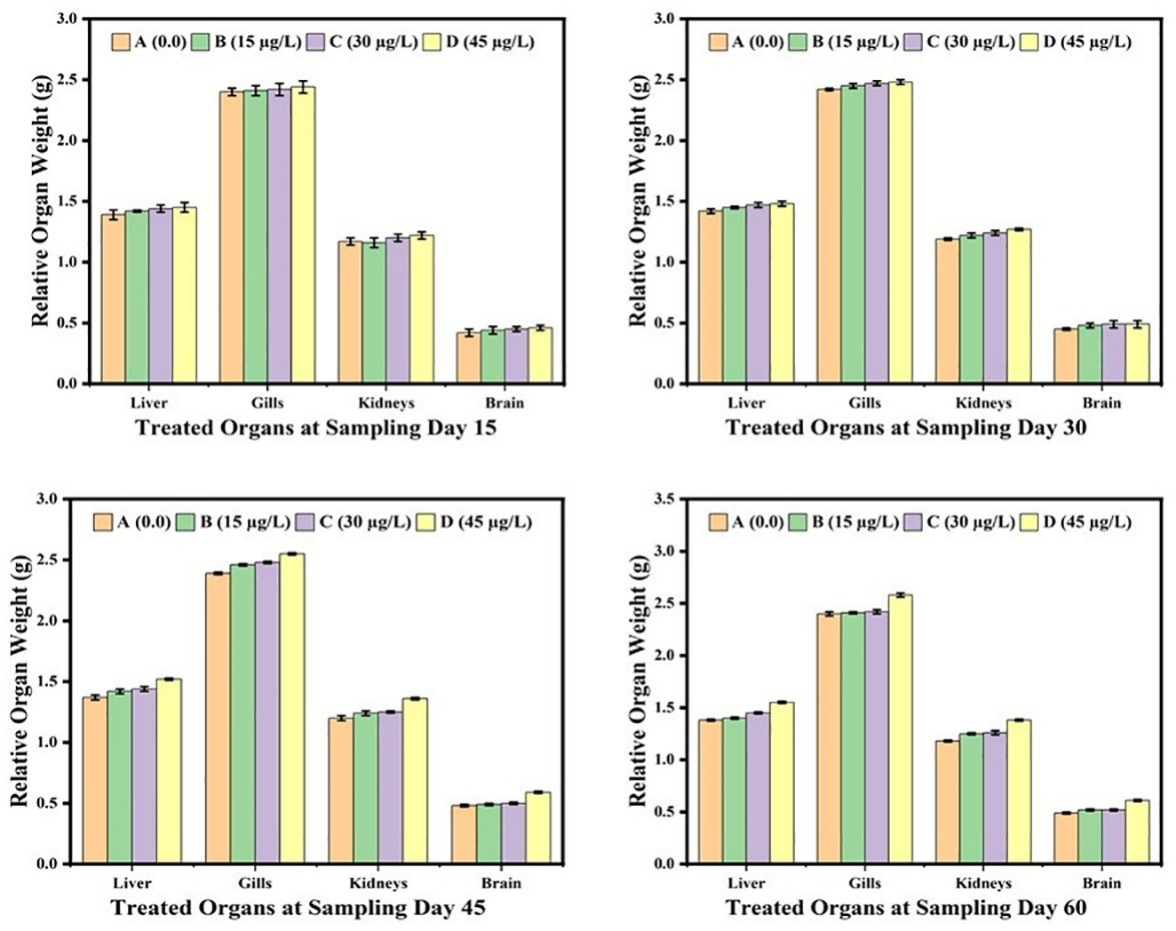
Relative weights (g) of different organs of fish exposed to different concentrations of lindane at different time intervals. The fish exposed with highest concentration (45μg/L) of lindane showed a significant (p<0.05) increase in the relative weights of liver, kidneys and gills compared to control group at the later stage of the study i.e. at days-45 and 60. The weight of brain was not affected by lindane toxicity during the course of experiment. Group A = control, B = 15μg/L, C = 30μg/L, D = 45μg/L of lindane.

The results showed that the body weight of the fish was not affected by low concentrations (15μg/L, 30μg/L) of lindane. While the body weight of the fish exposed to high concentration (45μg/L) of lindane, although initially remained unchanged but was reduced significantly (p<0.05) at day-60 of the trial in comparison to the control group (Fig 1). Compared to 15μg/L and 30μg/L concentrations of lindane that not significantly affected but at higher concentration (45μg/L) of lindane, the absolute (Fig 2) and relative (Fig 3) weights of visceral organs i.e. liver, kidneys and gills increased significantly (p<0.05) at days-45 and 60 of the study. There found no significant distinctions in the relative weight of brain between treated and untreated groups under the effect of any concentration of lindane, even at later days of experiment i.e. 45 and 60 days of the study (Fig 3).

### Hematological and serum biochemical analyses

**Fig 4:**
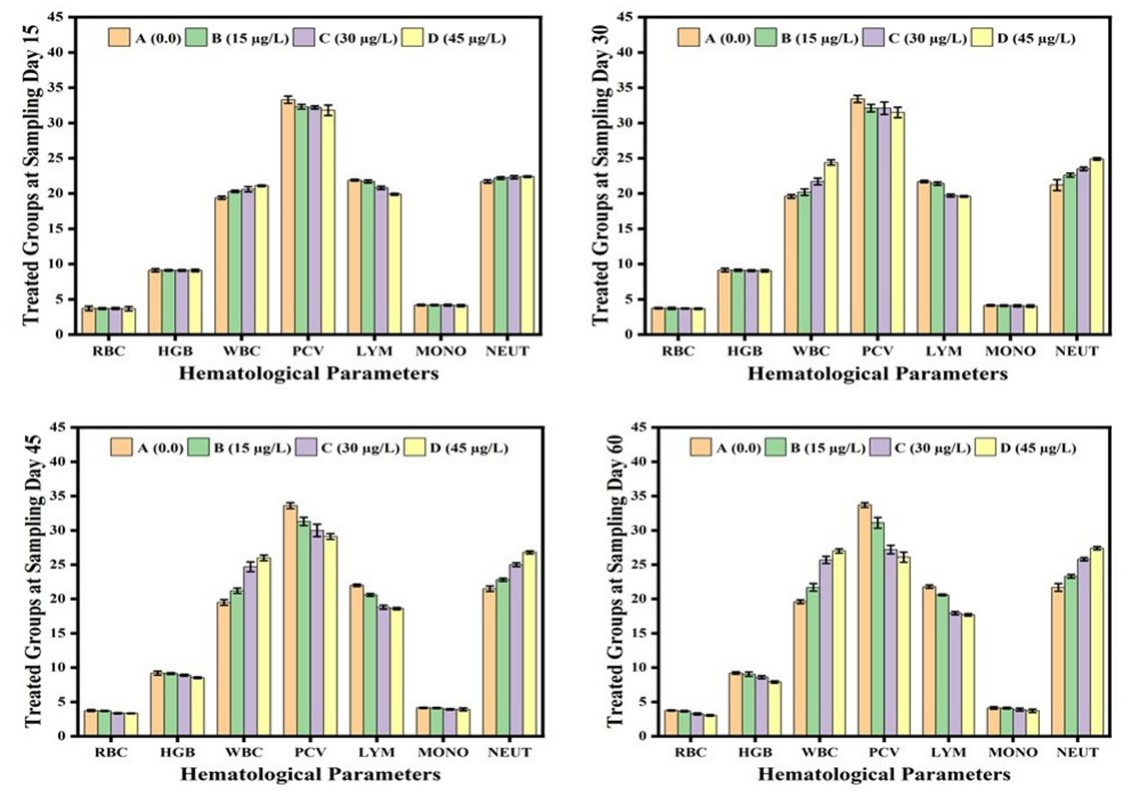
Hematological parameters of fish exposed to different concentrations of lindane. The fish exposed to higher concentrations (30μg/L and 45μg/L) of lindane showed neutrophilic leukocytosis while a significant (p<0.05) decrease in Hb level, RBCs, lymphocytes and monocytes count and PCV up-to the end of the experiment. Group A = control, B = 15μg/L, C = 30μg/L, D = 45μg/L of lindane.

**Fig 5:**
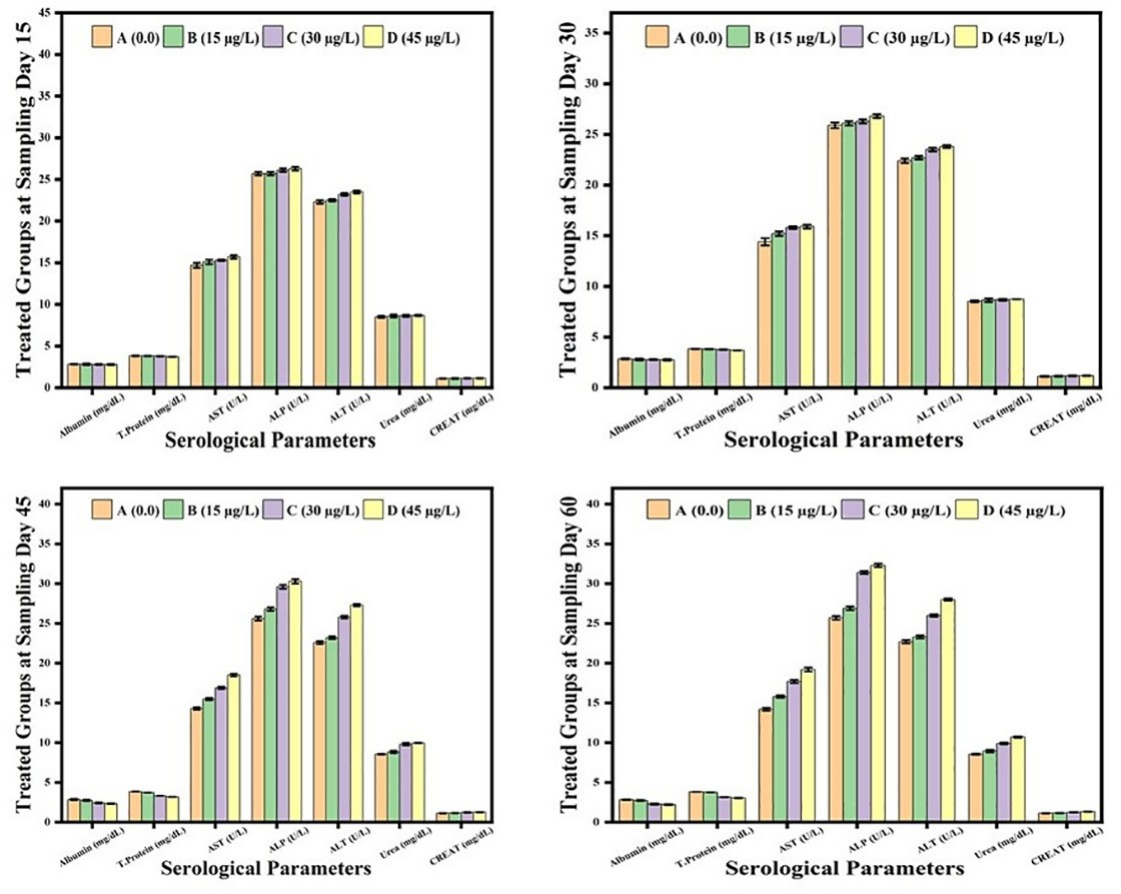
Serum biochemical profile of fish exposed to different concentrations of lindane. The level of serum albumin and total proteins substantially decreased while the amount of hepatic enzymes (ALT, AST and ALP) and renal profile (urea and creatinine) was significantly increased at higher lindane concentrations (30μg/L and 45μg/L) at days-45 and 60 of the experiment. Group A = control, B = 15μg/L, C = 30μg/L, D = 45μg/L of lindane.

The hematological and serum biochemical parameters remained unaffected by 15μg/L of lindane. These values also remained unaffected at the start of the experiment but at days-45 and 60, the fish exposed to 30μg/L and 45μg/L of lindane showed a significant decrease in Hb level, RBCs, lymphocytes and monocytes count and hence also the PCV (Fig 4). Contrarily, the fish exposed to these two concentrations of lindane exhibited a significant higher number of DLC (neutrophilic leukocytosis) at days-30, 45 and 60 of the experiment (Fig 4). At days-45 and 60 of the experiment, the level of serum albumin and total proteins substantially decreased in the fish exposed to 30μg/L and 45μg/L of lindane compared to unexposed fish (Fig 4). While the amount of ALT, AST and ALP (liver profile) and urea and creatinine (renal markers) was significantly increased at higher lindane concentrations (30μg/L and 45μg/L) at days-45 and 60 of the experiment (Fig 5).

### Genotoxicity of erythrocytes

**Fig 6:**
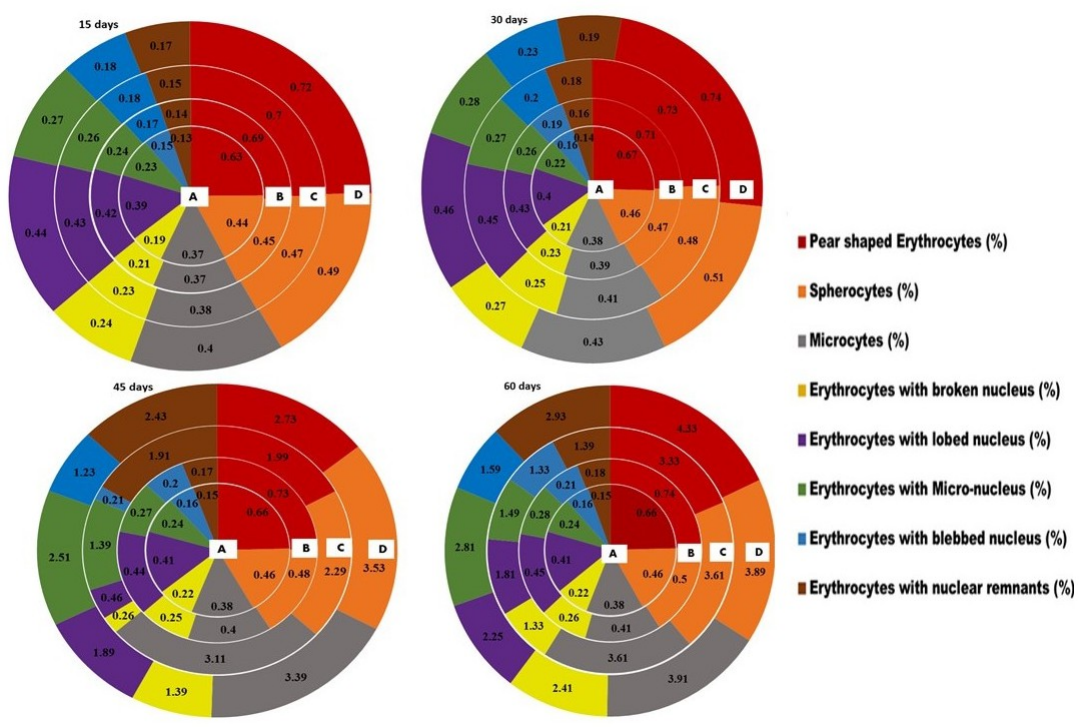
Frequency (%) of morphological changes and nuclear alterations (%) in RBCs of fish exposed to different concentrations of lindane. The fish exposed to 30μg/L and 45μg/L of lindane showed increased rate of modified pear-shaped erythrocytes, leptocytes, spherocytes and microcytes. The nuclear changes like lobed, blebbed or notched nuclei, micronucleus, vacuolated, fragmented, broken nuclei and nuclear remnants in the erythrocytes were found to be significantly higher in the fish exposed to 30μg/L and 45μg/L concentrations at days-45 and 60 of the trial compared to control group. Group A = control, B = 15μg/L, C = 30μg/L, D = 45μg/L of lindane.

**Fig 7:**
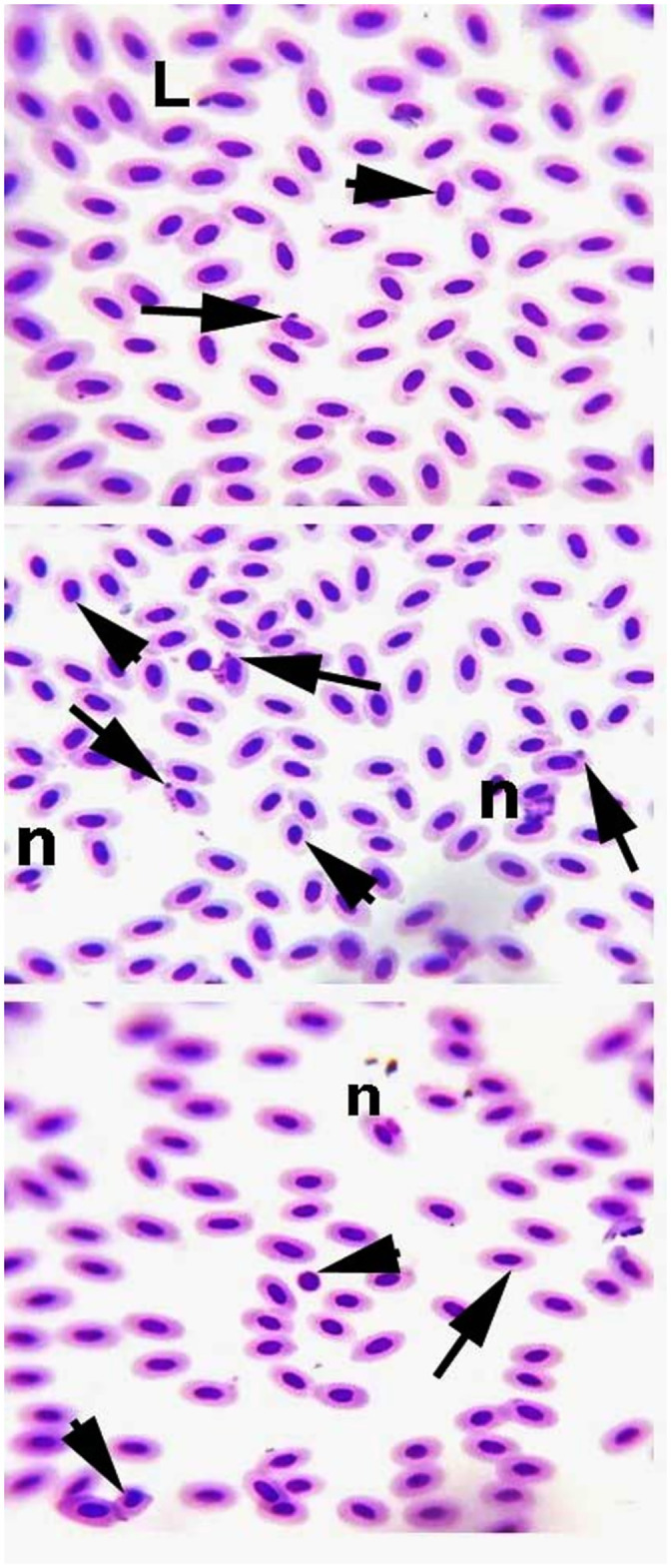
Blood smear exhibiting various morphological alterations in the nuclei of RBCs of the fish exposed to different concentrations of lindane. The erythrocytes having lobed nuclei (L), erythrocyte with micro nucleus (arrow), spherocytes (arrow head), nuclear remnants (n) or blabbed nucleus were significantly increased in the fish exposed to 30μg/L and 45μg/L lindane at the end of the experiment (day 60). Geimsa stain, Magnification: 1000X.

The fish exposed to 30μg/L and 45μg/L lindane showed increased rate of various morphological abnormalities in RBCs, while the lower concentration not caused any significant effects. The percentile rate of erythrocytes (modifiedpear shaped), leptocytes, spherocytes and microcytes (Fig 6) was significantly higher in the fish exposed to 30μg/L and 45μg/L concentrations at days-45 and 60 of the trial compared to the control group. Similarly, the percentile rate of various nuclear changes like lobed, blebbed or notched nuclei, micronucleus, vacuolated, fragmented, broken nuclei and nuclear remnants in the erythrocytes were significantly increased in the fish exposed to 30μg/L and 45μg/L of lindane compared to the control group. At the end of the experiment (day 60), the frequency of erythrocytes with lobed nuclei, erythrocytes with micro or blabbed nucleus was also found to be significantly higher in the fish exposed to all concentrations of lindane compared to the control (Fig 7).

### Parameters of oxidative stress and anti-oxidative enzymes

**Fig 8:**
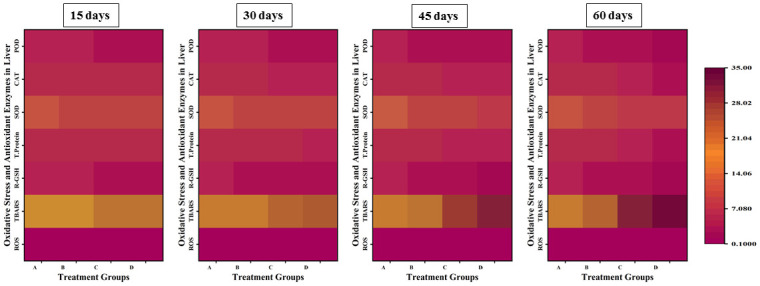
Oxidants and antioxidant biomarkers in the liver of fish exposed to different concentrations of lindane. ROS and TBARS showed a significant increase while CAT, SOD, POD and GSH contents were found to be significantly decreased in the liver of fish with an increase in the concentration and exposure time of lindane. Group A = control, B = 15μg/L, C = 30μg/L, D = 45μg/L of lindane.

**Fig 9:**
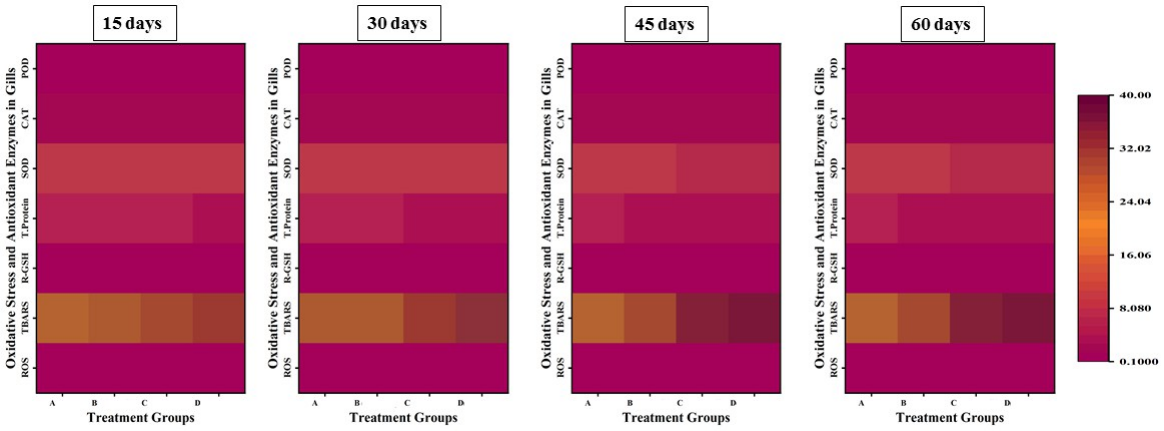
Oxidants and antioxidant biomarkers in the gills of fish under the effect of different concentrations of lindane. There found an increase in ROS and TBARS and a decrease in CAT, SOD, POD and GSH contents with an increase in the concentration of lindane towards the end of the experiment. Group A = control, B = 15μg/L, C = 30μg/L, D = 45μg/L of lindane.

**Fig 10:**
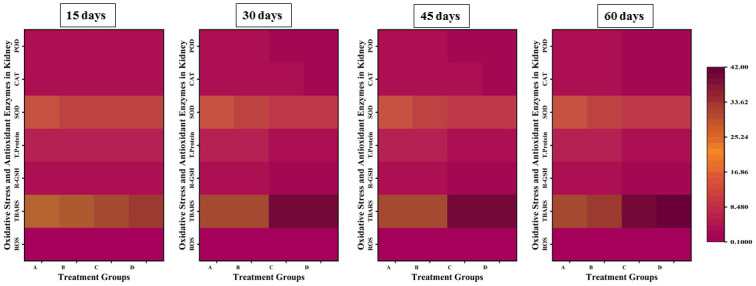
Oxidants and antioxidant biomarkers in the kidneys of fish exposed to different concentrations of lindane. With an increase in the concentration of lindane, the ROS and TBARS were significantly increased while CAT, SOD, POD and GSH were significantly decreased up-to the end of the experiment. Group A = control, B = 15μg/L, C = 30μg/L, D = 45μg/L of lindane.

**Fig 11:**
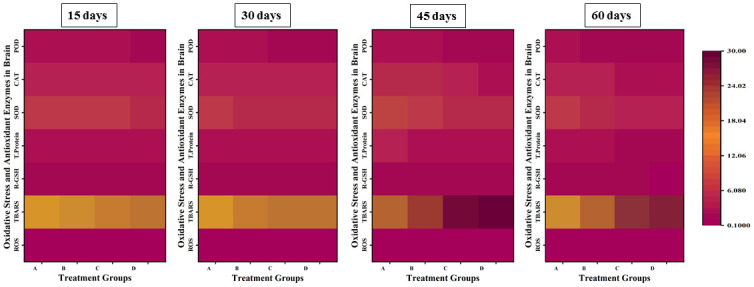
Oxidants and antioxidant biomarkers in the brain of fish exposed to different concentrations of lindane. The increased concentration of lindane leads to a significant increase in the ROS and TBARS values while there observed a significant decrease in the quantity of anti-oxidant enzymes in the brain of fish. Group A = control, B = 15μg/L, C = 30μg/L, D = 45μg/L of lindane.

The parameters of oxidative stress; ROS and TBARS showed a significant increase in the values with an increase in the concentration and exposure time of lindane, while there was a significant decrease in the levels of anti-oxidative enzymes; CAT, SOD, POD and GSH in liver (Fig 8), gills (Fig 9), kidneys (Fig 10) and brain (Fig 11) of *Labeo rohita* exposed to different concentrations of lindane.

### Histopathological observations

**Fig 12:**
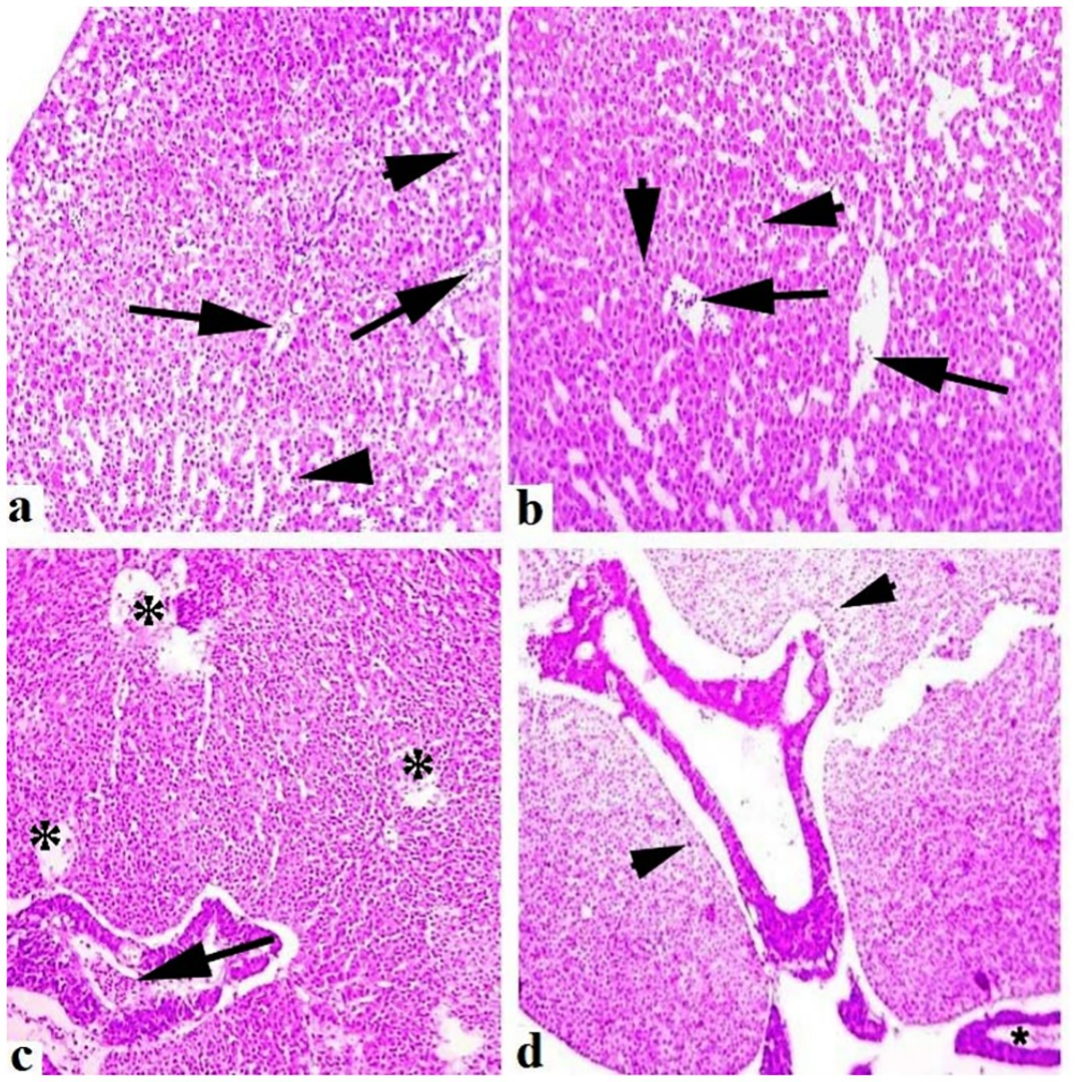
Histo-morphological changes in the liver of fish exposed to different concentrations of lindane. The affected liver exposed to 30 μg/L lindane at days-45 (a) and 60 (b) exhibited congestion, hemorrhage, nuclear pyknosis (arrow heads), karyorrhexis, karyolysis, binucleated hepatocytes, nuclear hypertrophy, eccentric nuclei (thin arrows) and inflammatory exudate (thick arrows). Liver sections at days-45 (c) and 60 (d) exhibited inflammatory exudates and disorganization of hepatocytes (*), hemorrhages (thick arrow), pyknosis (arrow heads) and eccentric hepatocyte (thin arrow) in the fish exposed to higher concentration (45μg/L) of lindane. Hematoxylin and Eosin stain, magnification: 400X.

**Fig 13:**
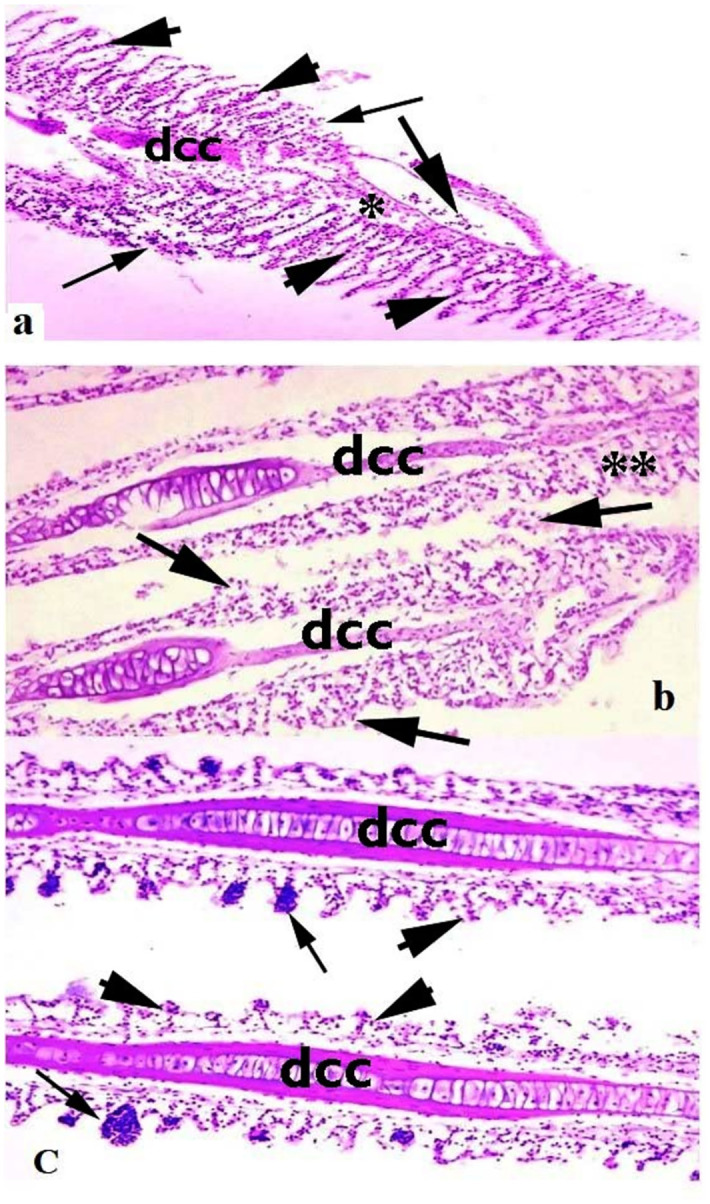
Histo-pathological changes in the gills of fish exposed to different concentrations of lindane. The gills of the fish exposed to 30 μg/L lindane (a) at days-45 and 60 exhibited different microscopic abnormalities like lamellar disorganization (*), necrosis of the lamellar epithelial cells (arrow heads), lamellar atrophy, fragmentation of primary lamellae, fusion of lamellae, congestion and deterioration of cartilaginous cores (dcc) and inflammatory material (arrow), b) detachment of lamellar epithelium (arrows), deterioration of cartilaginous cores (dcc) and fragmentation of secondary lamellae (**) at day-45 in the fish exposed to higher concentration (45μg/L) of lindane, c) lamellar atrophy and fusion (arrow heads), aneurysm (arrow) and deterioration of cartilaginous cores (dcc) were also observed in various sections of gills in the fish exposed to higher concentrations (45μg/L) of lindane at day-60. Hematoxylin and Eosin stain, magnification: 400X.

**Fig 14:**
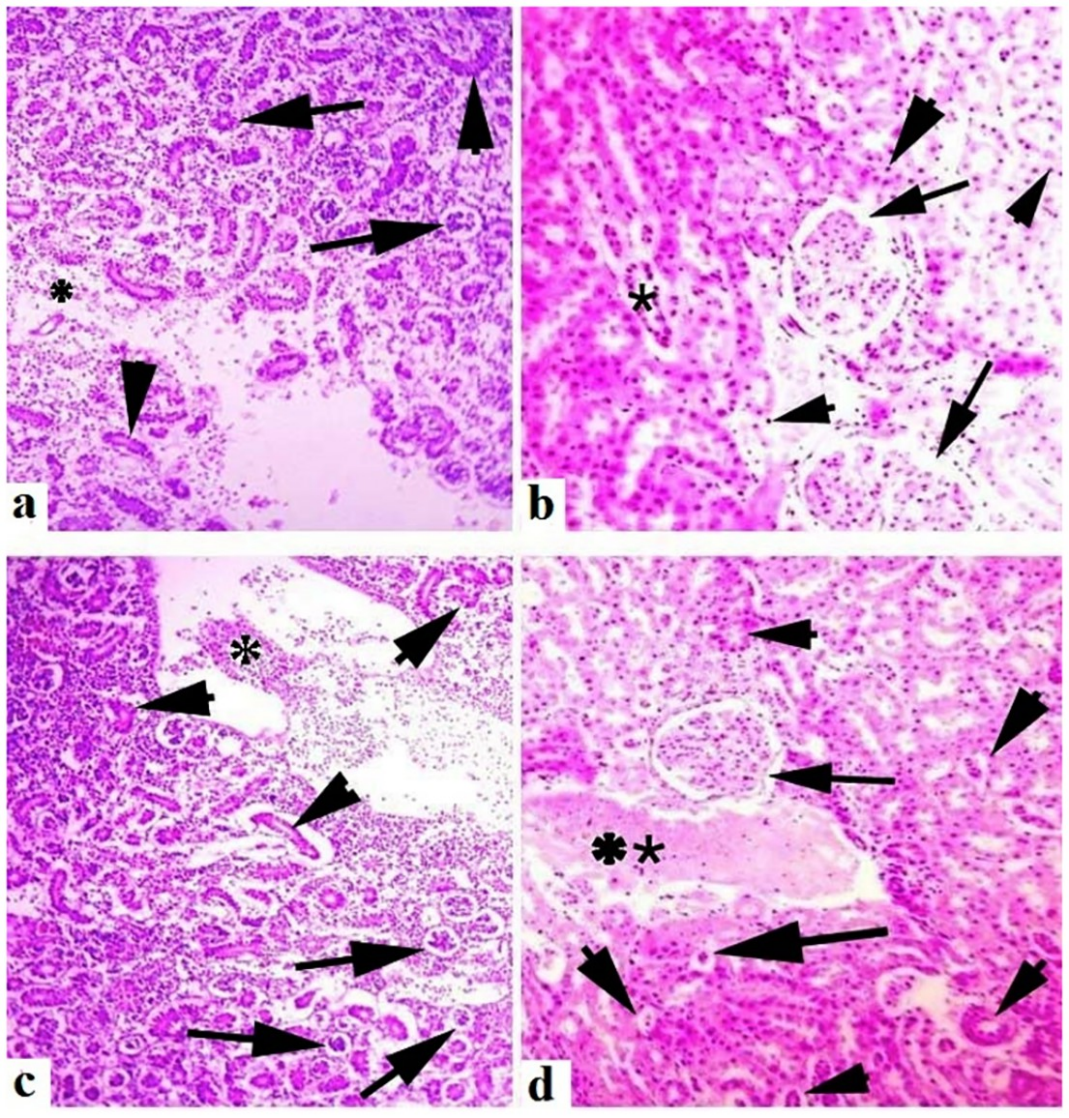
Histo-pathological changes in the kidneys of fish exposed to different concentrations of lindane. Kidneys (a) showing elevated Bowman’s space (arrows), degeneration and disorganization of renal tubules (arrow heads) and inflammatory cells (*) at day-45 while kidneys at day 60 (b) indicating nuclear pyknosis (arrow heads), glomerular deterioration (*) and elevated Bowman’s space (arrows) in the fish exposed to 30μg/L of lindane. Different sections of kidneys at days-45 (c) and 60 (d) are showing disintegration and deterioration of renal tubules (arrow heads), elevated Bowman’s space (arrows) and extensive inflammatory exudate (asterisks) in the fish exposed to higher concentration (45μg/L) of lindane. Hematoxylin and Eosin stain, magnification: 400X.

**Fig 15:**
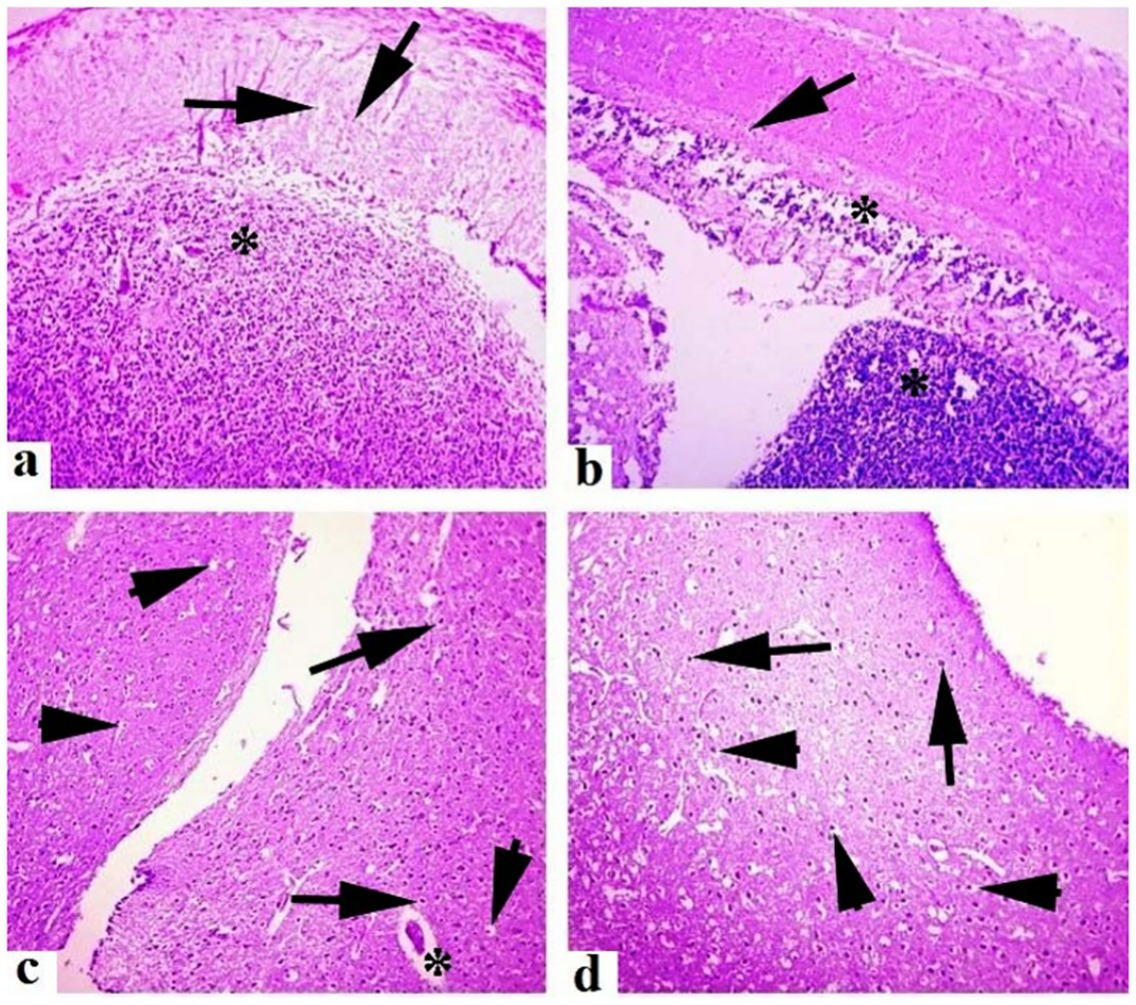
Histo-pathological changes in the brain of fish exposed to different concentrations of lindane. The brain of fish exposed to lindane (30μg/L) at days-45 (a) and 60 (b) showed degenerated neurons (arrows) and microgliosis (asterisk) while at days-45 (c) and 60 (d), the brain of the fish exposed to 45μg/L exhibited inflammatory exudate (*), eccentric nuclei of neurons (arrow heads), atrophy and necrosis of neurons (arrows). Hematoxylin and Eosin stain, magnification: 400X.

**Fig 16:**
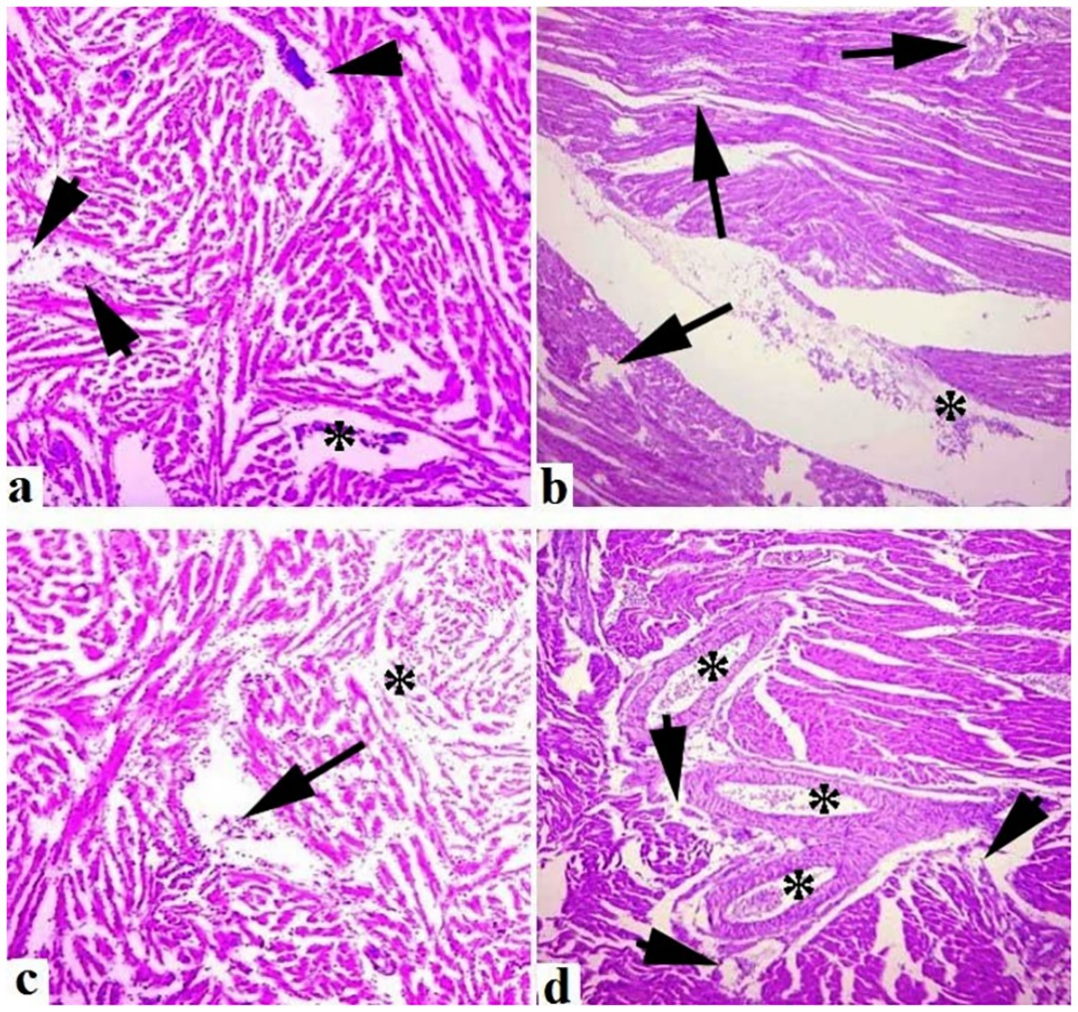
Histo-pathological changes in the heart of fish exposed to different concentrations of lindane. Microscopic abnormalities in the heart section, a) neutrophilic infiltration (arrow heads) and inflammatory exudate (*), b) damaged cardiac muscle fibers (arrows) and neutrophilic myocarditis (*) at days-45 and 60 in the fish exposed to 30μg/L of lindane, c) heart section showing neutrophilic infiltration, inflammatory exudate (arrow) and damaged cardiac muscle fibers (*) at day-45, d) extensive inflammatory exudate including hemorrhages (*) and necrosis of cardiac muscle fibers (arrow heads) was observed at day-60 in the fish exposed to higher concentration (45μg/L) of lindane. Hematoxylin and Eosin stain, magnification: 400X.

Medium to extreme histo-pathological anomalies in the liver like congestion, ceroid production, hemorrhage, nuclear pyknosis, karyorrhexis, karyolysis, binucleated hepatocytes, nuclear hypertrophy, eccentric nuclei and vacuolar disintegration were observed at days-45 and 60 of the experiment. Numerous histo-pathological defects in the fish liver exposed to high concentrations (30μg/L and 45μg/L) of lindane showed vacuolar disintegration, karyorrhexis, karyolysis on days-45 and 60 of the experiment (Fig 12). Severe histo-pathological abnormalities such as lamellar disorganization, necrosis of the lamellar pillar, lamellar atrophy, fragmentation of primary lamellae, curling of secondary lamellae, fusion of lamellae, extreme congestion and deterioration of cartilaginous cores and telangiectasia were observed in various parts of gills of the fish exposed to high concentrations (30μg/L and 45μg/L) of lindane. At days-45 and 60 of the experiment, curling and uplifting patterns in the epithelial cells of secondary lamellar were generally found in these groups (Fig 13). In the kidneys of all the treated groups, mild to moderate histo-pathological abnormalities like nuclear pyknosis, glomerular deterioration, congestion, elevated Bowman’s space, atrophic cells, edema, tubular epithelial disintegration, melano-macrophage aggregation and lumen atrophy were observed. Major histo-pathological alterations were apparent in the kidneys of fish in group B at days-45 and 60 of the experiment like degradation of glomeruli, increased bowmen’s space and necrosis of simple cuboidal epithelial cells of renal tubules (Fig 14). Microscopic observations of various sections of brain of treated fish exhibited the pathological alterations like inter- and intra-cellular edema, congestion, necrosis and cytoplasmic vacuolization at days-45 and 60 of the experiment. Moreover, degenerated neurons in cerebellum, microgliosis, necrosed neurons, lipofuscin deposition, inflammation in the cells and severe hemorrhage were observed in the groups exposed to 30μg/L and 45μg/L at days-45 and 60 of the trial (Fig 15). The histo-pathological alterations in the heart of fish exposed to higher levels of lindane (30μg/L and 45μg/L) were neutrophilic infiltration, necrosed cardiac myocytes, inflammation, edema, neutrophilic myocarditis, hemorrhages and deposition of fibrin were observed in the fish exposed to lindane (30μg/L and 45μg/L) at days-45 and 60 of the experiment (Fig 16).

## Discussion

Over the last few years, the extensive applications of lindane led to severe effects to the biota and hence the drug has now been considered as one of the most harmful POPs [27, 28]. Its chemical persistence and lipophilicity allows it to be biomagnified in the food chains [68]. Moreover, lindane is associated with various adverse health effects like immuno-suppressive, pro-inflammatory, carcinogenic, oxidative, neurotoxic and hormone-disrupting effects along with seizures and death in severe cases [24, 25]. Therefore, a prolonged monitoring and evaluation of the potential toxicity of lindane due to long-term exposure of its low concentrations to the land and water organisms is incredibly important in order to lessen its public health effects. In the current study, we have exposed the fish with quite low (sub-lethal) concentrations of lindane, in order to investigate the effects of minute concentrations of the drug that do not cause the death of the fish but might impart severe health effects. It has been found in the current investigation that all these concentrations not showed any apparent clinical signs and amongst the three tested concentrations, the lowest concentration (15μg/L) of lindane also had only negligible physico-chemical effects during the whole course of the study. While, the two high concentrations (30μg/L and 45μg/L) although had only mild effects at earlier stage of the experiment (at days-15 and 30) but showed significant aberrations in different parameters at the later stages of the study i.e. at days-45 and 60, in a concentration and time dependant manner.

In our study, we have observed significantly higher morphological ailments in RBCs in the treated fish as like expanded structural anomalies of erythrocytes including uncharacteristic shapes, vacuolation, invagination of membrane and fragmentation were observed in *Channa punctate* exposed to different concentrations of lindane [69]. In another report, greater strength of nuclear anomalies like nuclear aberrations of erythrocytes, micronuclei, terminal nucleus, extended and swollen nucleus and karyo-pyknotic were observed in *Barbonymus gonionotus* exposed to 1/10^th^ and 1/2 of LC_50_ concentrations of profenofos [70]. Moreover, lindane caused severe morphological deformities due to acute toxicity in *Barbus sophore*, *Clarius batrachus*, *Barbus goriontus*, *Cyprinus carpio*, *Tilapia mossambica* [71], *Corydoras paleatus* and *Jenynsia multidentata* [72]. In the current study, we have observed increased absolute and relative organ weights of all the visceral organs (gills, liver, heart and kidney) except the decrease in brain weight in fish. Except the few previous reports on relative organ weight of *Hypophthalmichthy snobilis* [73], *Labeo rohita* [16, 74], *Oreochromis niloticus* [75], Oreochromis sp. [76], cockerels [57] and rabbits [77] exposed to different toxicants, little is known about the effects of lindane on body tissues of aquatic and non-aquatic organisms. In the current study, the increase in absolute and relative weights of different visceral organs is related to inflammatory reactions in these organs, leading to accumulation of inflammatory exudate in the interstitial tissue spaces that ultimately cause an increase in the weight of the organ. Similarly, increased organ weight in *Labeo rohita* due to pyriproxyfen [74] and severe abnormalities in the weight of various tissues in different mammals due to malathion toxicity has also been recorded [78]. As like lindane, other organo-chlorine pesticides like thioacetamide, chlordane, endosulfan, diazion and dicofol have also found to cause potential toxic effects on hepatic, nervous, renal and reproductive tissues in rat, mice, fish and quail [23, 79–81]. Anti-oxidant enzymes are the biomarkers for inflammatory response of a cell due to free radicals [5, 75, 82–84] and the levels of anti-oxidant enzymes have been found to be inversely correlated with the toxicity of different organo-chlorine pesticides in *Cyprinus carpio* [85] that cause inactivation [23] or reduction in the level of anti-oxidant enzymes [86]. In the same context, a significant decrease has been found in the levels of anti-oxidative enzymes; CAT, SOD, POD and GSH in serum and the selected visceral organs of the fish in the current study. On the other hand, there observed a rise in the oxidants (ROS and TBARS) that might be due to mitochondrial dysfunction and an increase in the production of free radicals [87, 88]. It has been previously noted that even a single sub-lethal dose of endosulfan caused a high induction in ROS and lipid peroxidation with depletion in the level of natural anti-oxidants. It was thus demonstrated that an imbalance in the redox status of cells and the role of mitochondrial distress causally related to the cellular oxidative stress and thus an induction in ROS on endosulfan treatment [86]. Therefore, the same is implicated in our current investigation that the toxicity stress of lindane leads to significantly increased levels of oxidative stress factors and a decrease in the anti-oxidative enzymes at the same time, in the blood and various tissues.

In the current study, we have observed a lower Hb concentration, decreased RBCs count and PCV that might be due to rapid Hb oxidation and destruction and hemolysis of erythrocytes due to toxicity effects [41, 89]. An increased number of WBCs and neutrophils were observed at higher concentrations of lindane that might be attributed to immunological reactions expressed by tissue injury in the exposed fish. Similar to our observations, a significant reduction in RBCs count and Hb, hematocrit were also observed in *Etroplus maculates* [47] and *Aspidoparia morar* [53] fish exposed to sub-lethal concentrations of lindane. Simailarly, abnormalities in the hematological parameters were observed in *Heteropneustes fossilis* [90], *Clarias gariepinus* [9, 91, 92] and rodents [93] exposed to different toxicants. Similarly, lindane has been found to cause adverse effects on serum biochemical profile like ALT, AST and ALP increased significantly in association with lindane-induced stress resulting in an impairment in growth performance and fish well-being. Elevated levels of urea and creatinine in the liver and kidney of the affected fish might be reflecting a disruption in the renal filtration mechanism and damage to the kidneys and liver tissues of the fish exposed to lindane. The adverse changes in LDH, AST, ALT, cholinesterase, glutathione peroxidase and catalase enzymatic activity has also been reported previously in *Cyprinus carpio* [45] and *Oncorhynchus mykiss* [94] exposed to chlorpyrifos and lindane, respectively. Moreover, serum biochemical alteration were also observed in *Oncorhynchus mykiss* [95], *Oreochromis niloticus* [96], *Channa punctatus* [97] and *Cyprinus carpio L*. [98] exposed to different toxicants.

The alterations in the serum profile are also related to the histo-pathological changes in the visceral organs, likewise gills, liver and renal tissue aberrations have been observed in our study, as like previously found in *Etroplus maculates* fish exposed to lindane [47]. We have observed the lesions in the hepatic tissue like congestion, decreased cytoplasmic volume, vacuolar deterioration, expanded sinusoidal space, hepatocyte karyolysis and necrosis in the liver of fish subjected to higher concentrations of lindane. Similar changes in the liver histology like cytoplasmic vacuolation, nuclear hypertrophy, internal bleeding and pycnotic region and cytoplasmic vacuolation in *Heteropneustes fossilis* exposed to envoy 50 SC have been reported earlier [99]. Moreover, hepatocytes clusters and cloudy swelling, hypertrophy, disorientation and bile duct obstruction were found in *C*. *carpio* [100]. Histo-pathological lesions like edema, ceroid formation, glomerular deterioration, reduced Bowman’s space, tubule congestion atrophy and lumen atrophy were seen in the kidneys of treated fish as like decreased nephron count, glomerular lesions and decreased glomerular filtration rate were observed in the kidneys of *Salmo salar* [101], rainbow trout [102], *Labeo rohita* [16] and *Aristichthys nobilis* [59] exposed to different toxicants. Similar findings observed in other fish like *Heteropneustes fossilis* [103], tilapia [104, 105] and *Catla catla* [59] exposed to toxins were necrosis, vacuolation, accumulation of melano-macrophages, ossification, blood congestion, cellular rupture, nuclear hypertrophy degeneration, pyknotic nucleus and lumen reduction. It has been shown that histo-pathological lesions in the gills are an effective sign for screening the impact of various pollutants in the freshwater environment since the gills are the first organs in contact with water pollutants, to which the contaminants penetrate. The histo-pathological lesions in gills found in the current study include atrophied lamellae, lamellar fusion, lamellae uplift, obstruction and disorganization of primary and secondary lamellae. Similarly, lindane also caused necrosis, lamellar deformation, epithelium degradation, vacuolation, hyperplasia, tubular alteration, neoplasia, hemocyte invasion, hypertrophy, pyknosis and other histological aberrations in *C*. *Fluminea* [106]. The depletion of total protein contents in the fish muscles might be due to energy diversion [107, 108] or due to leaching of soluble components particularly water proteins [109, 110] due to toxic stress of lindane. Moreover, the decrease in protein, fat and carbohydrate contents and increased moisture contents were observed in *C*. *catla*, *L*. *rohita* and *C*. *mrigala* under the effects of different sub-lethal concentrations of profenofos [111].

Hence, the results of the current study indicate that lindane at sub-lethal concentration changes the hematological and biochemical parameters of *L*. *rohita* that could be used to determine the physiological condition of the fish and to detect the adverse effects of lindane in the aquatic environment. The proximate composition of fish meat and the histo-pathological studies of different tissues further confirmed the degree of damage in vital organs of the fish that shows that lindane is capable to cause severe damage to the vital organs (brain, gills, lungs, heart and liver) of the fish leading to altered enzyme levels that could potentially influence the health and reproduction of fish and thus, could ultimately affect the humans on consuming these affected fish. Hence, it is quite obvious that there should not be an extensive use of pesticides and their discharge in the canal and river water should be strictly restricted that are severely detrimental for natural inhabitants and other food species of freshwater environments.

## Conclusion

To the best of our knowledge, the current study is the first detailed and comprehensive investigation of toxic effects of lindane at sub-lethal concentrations on complete hemato-biochemical profile and histo-pathological parameters of several organ tissues of *Labeo rohita*. The results of our experimental trial showed that lindane causes severse toxic effects on fresh water fish influencing blood biochemistry, histopathology and induction of oxidative stress in a concentration and time dependent manner. The parameters could be used to determine the physiological condition of the fish and to detect the adverse effects of lindane in the aquatic environments. Hence, it is obvious that the extensive use of pesticides should be restricted and their exposure to canal and river water is severely detrimental for natural inhabitants and food species of freshwater environment.
